# Uniform single atomic Cu_1_-C_4_ sites anchored in graphdiyne for hydroxylation of benzene to phenol

**DOI:** 10.1093/nsr/nwac018

**Published:** 2022-02-11

**Authors:** Jia Yu, Changyan Cao, Hongqiang Jin, Weiming Chen, Qikai Shen, Peipei Li, Lirong Zheng, Feng He, Weiguo Song, Yuliang Li

**Affiliations:** Beijing National Laboratory for Molecular Sciences, CAS Research/Education Center for Excellence in Molecular Sciences, Key Laboratory of Molecular Nanostructures and Nanotechnology, Institute of Chemistry, Chinese Academy of Sciences, Beijing 100190, China; University of Chinese Academy of Sciences, Beijing 100049, China; Beijing National Laboratory for Molecular Sciences, CAS Research/Education Center for Excellence in Molecular Sciences, Key Laboratory of Molecular Nanostructures and Nanotechnology, Institute of Chemistry, Chinese Academy of Sciences, Beijing 100190, China; University of Chinese Academy of Sciences, Beijing 100049, China; Beijing National Laboratory for Molecular Sciences, CAS Research/Education Center for Excellence in Molecular Sciences, Key Laboratory of Molecular Nanostructures and Nanotechnology, Institute of Chemistry, Chinese Academy of Sciences, Beijing 100190, China; University of Chinese Academy of Sciences, Beijing 100049, China; Beijing National Laboratory for Molecular Sciences, CAS Research/Education Center for Excellence in Molecular Sciences, Key Laboratory of Molecular Nanostructures and Nanotechnology, Institute of Chemistry, Chinese Academy of Sciences, Beijing 100190, China; University of Chinese Academy of Sciences, Beijing 100049, China; Beijing National Laboratory for Molecular Sciences, CAS Research/Education Center for Excellence in Molecular Sciences, Key Laboratory of Molecular Nanostructures and Nanotechnology, Institute of Chemistry, Chinese Academy of Sciences, Beijing 100190, China; University of Chinese Academy of Sciences, Beijing 100049, China; Beijing National Laboratory for Molecular Sciences, CAS Research/Education Center for Excellence in Molecular Sciences, Key Laboratory of Molecular Nanostructures and Nanotechnology, Institute of Chemistry, Chinese Academy of Sciences, Beijing 100190, China; University of Chinese Academy of Sciences, Beijing 100049, China; Beijing Synchrotron Radiation Facility (BSRF), Institute of High Energy Physics, Chinese Academy of Sciences, Beijing 100049, China; Beijing National Laboratory for Molecular Sciences, CAS Research/Education Center for Excellence in Molecular Sciences, Laboratory of Organic Solids, Institute of Chemistry, Chinese Academy of Sciences, Beijing 100190, China; Beijing National Laboratory for Molecular Sciences, CAS Research/Education Center for Excellence in Molecular Sciences, Key Laboratory of Molecular Nanostructures and Nanotechnology, Institute of Chemistry, Chinese Academy of Sciences, Beijing 100190, China; University of Chinese Academy of Sciences, Beijing 100049, China; University of Chinese Academy of Sciences, Beijing 100049, China; Beijing National Laboratory for Molecular Sciences, CAS Research/Education Center for Excellence in Molecular Sciences, Laboratory of Organic Solids, Institute of Chemistry, Chinese Academy of Sciences, Beijing 100190, China

**Keywords:** single-atom catalysis, graphdiyne, copper, benzene oxidation, phenol

## Abstract

For single-atom catalysts (SACs), the catalyst supports are not only anchors for single atoms, but also modulators for geometric and electronic structures, which determine their catalytic performance. Selecting an appropriate support to prepare SACs with uniform coordination environments is critical for achieving optimal performance and clarifying the relationship between the structure and the property of SACs. Approaching such a goal is still a significant challenge. Taking advantage of the strong d-π interaction between Cu atoms and diacetylenic in a graphdiyne (GDY) support, we present an efficient and simple strategy for fabricating Cu single atoms anchored on GDY (Cu_1_/GDY) with uniform Cu_1_-C_4_ single sites under mild conditions. The Cu atomic structure was confirmed by combining synchrotron radiation X-ray absorption spectroscopy, X-ray photoelectron spectroscopy and density functional theory (DFT) calculations. The as-prepared Cu_1_/GDY exhibits much higher activity than state-of-the-art SACs in direct benzene oxidation to phenol with H_2_O_2_ reaction, with turnover frequency values of 251 h^−1^ at room temperature and 1889 h^−1^ at 60°C, respectively. Furthermore, even with a high benzene conversion of 86%, high phenol selectivity (96%) is maintained, which can be ascribed to the hydrophobic and oleophyllic surface nature of Cu_1_/GDY for benzene adsorption and phenol desorption. Both experiments and DFT calculations indicate that Cu_1_-C_4_ single sites are more effective at activating H_2_O_2_ to form Cu=O bonds, which are important active intermediates for benzene oxidation to phenol.

## Teaser

Cu single atoms anchored on graphdiyne with uniform Cu_1_-C_4_ single sites exhibit superior activity and selectivity in direct benzene oxidation to phenol with H_2_O_2_.

## INTRODUCTION

**Scheme 1.**
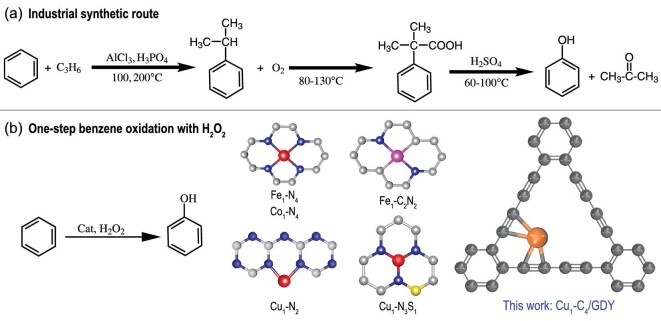
A comparison of the synthetic routes for phenol. (a) Industrial synthetic route and (b) one-step benzene oxidation with H_2_O_2_, with different metal single atom catalysts, as found in the literature [15,18,20–22] and in this work.

Phenol is an important industrial raw material for the production of various chemicals, including dichlorphenol A, phenolic resins, aniline and salicylamide [1]. Because of the rapid development of the electronic communications industry, the automobile industry and the construction industry in recent years, the demand for bisphenol A and phenolic resins, and thus the demand for phenol, has increased significantly. At the moment, there are two main methods for industrial phenol synthesis: the benzoic acid process and the cumene process, with the latter accounting for more than 95% of global phenol production [1]. Along with its success, the cumene process also suffers several disadvantages, including harsh reaction conditions (high temperature and pressure, strongly acidic conditions) and multistep reaction sequences, which result in a low yield of phenol (<5%) and serious environmental pollution (Scheme 1a). Thus, under mild reaction conditions, one-step direct oxidation of benzene to phenol, with green and economical hydrogen peroxide as the oxidant, is regarded as a promising route and has sparked intense research interest (Scheme 1b) [2]. Various novel heterogeneous catalysts, such as zeolites and transition metal nanomaterials, have been developed for this reaction in recent years, with many issues still to be resolved [3,4].

Single-atom catalysts (SACs) have been actively studied in the past decade due to their notable properties, such as 100% atom utilization, unsaturated coordination structure and particular electronic structure [5–14]. All of these characteristics give SACs superior activity and selectivity when it comes to producing target products in a variety of catalytic reactions. In the direct oxidation of benzene to phenol reaction, Fe, Co and Cu single atoms anchored on heteroatom doped-carbon supports were found to perform better than nanoparticles (Scheme 1b) [15–19]. The catalytic activity of these SACs, in particular, can be improved by modifying their coordinated structures, which affect the local geometric configuration and electronic structure of single atoms [20–22]. However, all the SACs reported above were fabricated through the high-temperature pyrolysis process, which was not only energy intensive but also made it hard to obtain a precise uniform coordinated single site. This is a common problem as well as a challenge for the current SAC research field and restricts a clear understanding of the influence of coordination structure on the performance of SACs [23,24].

Graphdiyne (GDY), a new two-dimensional periodic carbon allotrope with an atom-thick layer, is composed of sp-hybridized carbon atoms in diacetylenic and sp^2^-hybridized carbon atoms in benzene rings [25]. The unique alkyne-rich structure of GDY, in particular, makes it an ideal support for anchoring single atoms due to the uniformly distributed pores and large binding energies to metal atoms via the strong d-π interaction. Using these characteristics, Li and colleagues synthesized a variety of GDY-supported single metal atoms (Fe, Ni, Pd, Mo) with lower and even zero-valence states that demonstrated excellent activity and stability in electrocatalysis [26–30]. Compared with the conventional SACs, GDY-based SACs can be synthesized under mild conditions and have uniform well-defined metal_1_-C_4_ coordinated structures, providing an ideal model and opportunity to identify the active sites during catalysis and therefore determine the catalytic mechanism at the atomic level. However, there have been no reports about GDY-supported metal SACs for direct oxidation of benzene to phenol.

In this manuscript, we develop a facile approach to fabricating Cu single atoms anchored on GDY (denoted as Cu_1_/GDY) under mild conditions, e.g. 120°C in the air, within seconds. The formation of low-valence Cu^δ+^ (0 < δ < 1) with uniform Cu_1_-C_4_ single sites on GDY was confirmed by synchrotron radiation X-ray absorption spectroscopy (XAS), X-ray photoelectron spectroscopy (XPS) and density functional theory (DFT) calculations. Cu_1_/GDY demonstrated excellent catalytic performance for benzene oxidation to phenol using H_2_O_2_. The calculated turnover frequency (TOF) is ∼251 h^−1^ at room temperature and 1889 h^−1^ at 60°C, which is significantly higher than previously reported catalysts under the same reaction conditions. The surface of as-prepared Cu_1_/GDY is particularly hydrophobic and oleophyllic, which is advantageous for benzene adsorption and phenol desorption, resulting in high phenol selectivity (96%) despite a high benzene conversion of 86%. Both experiments and DFT calculations indicate that the Cu_1_-C_4_ single site can effectively activate H_2_O_2_ to form Cu=O bonds with a more stable O-Cu_1_-C_4_ single site in GDY, which are important active intermediates for benzene oxidation to phenol. Moreover, the calculated *d*-band position of a Cu single atom in O-Cu_1_-C_4_/GDY is much closer to Fermi level compared to those of reported Cu SACs with nitrogen coordination in graphene O-Cu_1_-Nx/G (x = 2, 3, 4), clarifying the intrinsic high activity of Cu_1_/GDY for benzene oxidation to phenol with H_2_O_2_.

## RESULTS AND DISCUSSION

### Synthesis and characterizations

**Figure 1.**
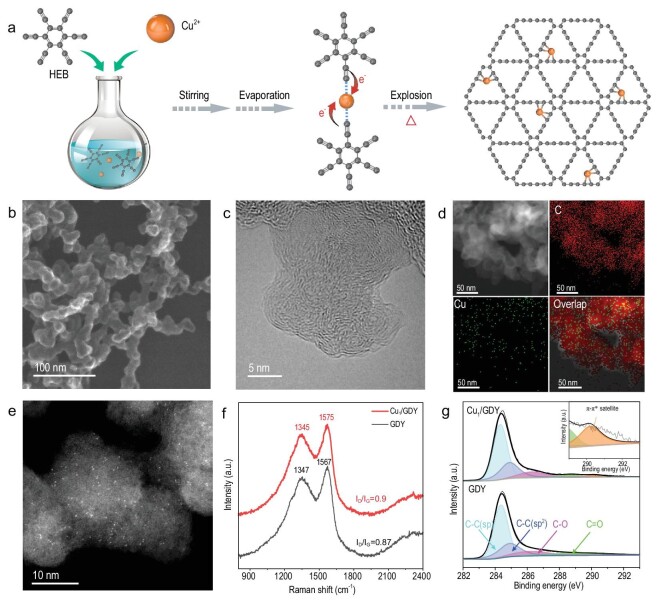
(a) Illustration of the synthesis procedures for Cu_1_/GDY. (b) SEM, (c) HRTEM, (d) energy dispersive spectroscopy (EDS) mapping and (e) AC HAADF-STEM images of Cu_1_/GDY. (f, g) Raman spectra and XPS spectra of GDY and Cu_1_/GDY.

Preparation of GDY-supported SACs was usually based on well-established reductive elimination reactions with metal catalysts. Typically, GDY monomers and metal cations form coordination complexes, and the metal centers accept electrons from the substrates, resulting in the formation of C-C bonds and low-valence metal centers *in situ* [31]. Zuo and Li *et al*. reported a novel explosion method for efficiently preparing GDY by directly heating hexaethynylbenzene (HEB) at 120°C in the air without the use of a metal catalyst [32]. Combining the above results and making use of the strong d-π interaction between abundant alkyne bonds in HEB and Cu^2+^, we proposed a coordinating-explosion reductive elimination approach to synthesizing Cu_1_/GDY. As shown in Fig. 1a, copper acetate is first added to a THF solution of HEB and stirred for 1 h. During this process, HEB can be deprotonated to some extent, and forms a coordination complex with Cu^2+^, resulting in an inner-sphere electron transfer between C-C triple bonds and Cu^2+^. After the solvent is removed in a vacuum, the solid light-yellow powder is transferred to a preheated conical flask (120°C) in air, where cross coupling occurs quickly, resulting in the formation of dark Cu_1_/GDY within seconds (Fig. S1). During the formation process, Cu^2+^ is reduced to low-valence Cu^δ+^ (0 < δ < 1) and trapped by electron-rich diacetylene units in the GDY framework through stereo-confinement. In comparison to the common high-temperature pyrolysis method for synthesis of transition metal SACs with heteroatom coordination (N, P or S), this method for synthesis of Cu_1_/GDY is simple and efficient under mild conditions. In GDY, a single Cu atom is coordinated with two diacetylenic atoms to form a uniform Cu_1_-C_4_ single site.

Typical scanning electron microscopy (SEM) and transmission electron microscopy (TEM) images (Fig. 1b and Fig. S2a and b) show that the obtained Cu_1_/GDY is composed of small irregular nanoparticles, which are similar to those of pure GDY without Cu^2+^ by the same explosion approach. The high-resolution TEM (HRTEM) image in Fig. 1c and Fig. S1c and d reveals an onion-like morphology of the nanoparticles with an interlayer distance of ∼3.66 Å, which is similar to pure GDY. Furthermore, no obvious Cu aggregates were found in the HRTEM image. The corresponding elemental mapping images (Fig. 1d) indicate that Cu species are distributed uniformly on the GDY support. Cu content in Cu_1_/GDY was measured to be ∼0.21 wt% through inductively coupled plasma atomic emission spectrometry (ICP-AES). To inspect Cu species, aberration-corrected high-angle annular dark-field scanning TEM (AC-HAADF-STEM) was used. Because of the different Z contrast, the isolated bright dots corresponding to individual Cu atoms could be seen in the GDY support, as shown in Fig. 1e.

Raman and XPS were used to characterize the structure of Cu_1_/GDY and the interaction between Cu atoms and the GDY support. As shown in Fig. 1f, two obvious peaks at ∼1340 cm^−1^ and 1570 cm^−1^ are observed in both pristine GDY and Cu_1_/GDY, corresponding to the D band and G band of sp^2^ carbon in aromatic rings, respectively. The band shifts to higher wavenumbers in Cu_1_/GDY when compared to pristine GDY, most likely due to interactions between Cu atoms and the GDY support [26,28]. Furthermore, the ratio of D- and G-band intensity (I_D_/I_G_) for Cu_1_/GDY (0.90) is slightly higher than that of pristine GDY (0.87), indicating that more defects and active sites are formed after anchoring Cu single atoms. Unfortunately, no obvious peak of −C≡C− vibration band at ∼2190 cm^−1^ is observed in pristine GDY and Cu_1_/GDY samples. This is likely because of the higher number of defects of GDY produced by the explosion method. In contrast, GDY prepared through the traditional liquid method usually has a significantly lower I_D_/I_G_ ratio (0.77 vs. 0.87). The X-ray powder diffraction pattern also confirms the low crystallization and many defects of as-prepared GDY, as only a broad peak centered at 23° is observed (Fig. S3). However, in their C 1s orbital XPS spectra (Fig. 1g), peaks assigned to sp-C (284.2 eV) and sp^2^-C (284.8 eV) are observed and the area ratios of sp-C to sp^2^-C are close to 2, indicating a well-maintained GDY skeleton even after loading Cu atoms. Moreover, compared with pristine GDY, an additional small peak at 290.2 eV, which attributes to the π-π^*^ transition, is observed in Cu_1_/GDY [26,28]. This indicates the presence of interactions between Cu atoms and the GDY support, following the above Raman results.

**Figure 2.**
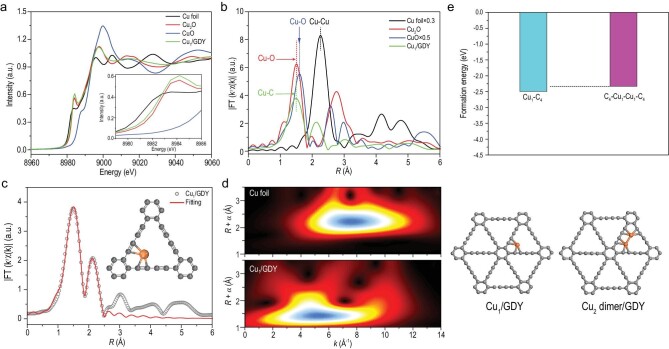
Synchrotron XAFS measurements of Cu_1_/GDY. (a) Cu K-edge XANES spectra of Cu_1_/GDY and reference samples. (b) Fourier transformed (FT) k^3^-weighted χ(k)-function of the EXAFS spectra for Cu K-edge. (c) Corresponding EXAFS fitting curve at R space, inset showing the schematic model. (d) Wavelet transforms for the k^3^-weighted EXAFS signals. (e) Formation energies of Cu_1_-C_4_/GDY and Cu_2_ dimer/GDY as produced by DFT calculations.

Synchrotron radiation XAS was performed to investigate the chemical state and coordination structure of Cu atoms in Cu_1_/GDY. As shown in Fig. 2a, the Cu K-edge X-ray absorption near edge structure (XANES) spectrum of Cu_1_/GDY is higher than Cu foil and lower than Cu_2_O, indicating that the Cu species are partially positively charged with low-valence (Cu^δ+^, 0 < δ < 1) due to the charge redistribution between Cu^0^ and GDY, or easy oxidation of Cu^0^ single atoms. The high-resolution Cu 2p^3/2^ XPS spectrum of Cu_1_/GDY (Fig. S4) shows the peak at 932.9 eV, which is higher than that of Cu^0^ (932.4 eV) but lower than that of Cu^1+^ (933.0 eV), also confirming the low-valence Cu^δ+^ species in Cu_1_/GDY. Fourier-transformed k^3^-weighted extended X-ray absorption fine structure (EXAFS) in R space revealed a single notable peak at 1.49 Å (without phase change), which can be attributed to the first coordination shell of the Cu-C bond in Cu_1_/GDY (Fig. 2b). The results of the EXAFS fitting are shown in Fig. 2c and Table S1. The result shows that Cu-C, with the first coordination shell at a distance of 1.92 Å, has an average coordination number of 4.0, demonstrating single Cu atoms are coordinated by four surrounding sp-C atoms. In particular, another obvious peak at 2.11 Å can also be observed, which is different from Cu-Cu (2.26 Å) in Cu foil. This distance can be matched well to the Cu-C with sp^2^-C of the benzene ring (as shown in the inset of Fig. 2c). As we know, synchrotron radiation XAS is very sensitive to local atoms. The presence of an obvious peak at 2.11 Å provides conclusive evidence that the Cu single atom is coordinated with the dialkyne of the GDY skeleton to form the uniform Cu_1_-C_4_ site. As confirmed by DFT calculations and reported in numerous related publications, this special configuration structure is an important feature of GDY support, to anchor metallic single atoms. The wavelet transform contour plots show only one intensity maximum at ∼4.5 Å, which can be assigned to Cu-C (Fig. 2d). No obvious Cu-Cu aggregate peak (compared with Cu foil) can be observed. A DFT calculation was further carried out to confirm the coordination structure of Cu atoms in Cu_1_/GDY. Figure 2e depicts the formation energies of Cu_1_-C_4_ and C_4_-Cu_1_-Cu_1_-C_4_ sites in the GDY matrix. It can be seen that Cu single atoms can be anchored by the diacetylene of GDY to maintain their stability, and that they are even more stable than Cu_2_ dimers. When the above results and discussions are combined, it is clear that Cu species exist as isolated single atoms and are coordinated with four carbon atoms in this Cu_1_/GDY sample, similar to other GDY-supported metal SACs in the literature.

Additionally, the as-prepared Cu_1_/GDY exhibits a typical type I gas adsorption isotherm (Fig. S5), consistent with the nature of the microporous structure of GDY. The Brunauer-Emmett-Teller (BET) specific surface area is calculated to be ∼349 m^2^ g^−1^, which is only smaller than that of pure GDY (398 m^2^ g^−1^). Such a large BET value can be attributed to the small size of Cu_1_/GDY nanoparticles, with many defects and Cu single atoms, which is also beneficial for catalysis.

### Catalytic performance for benzene oxidation

**Figure 3.**
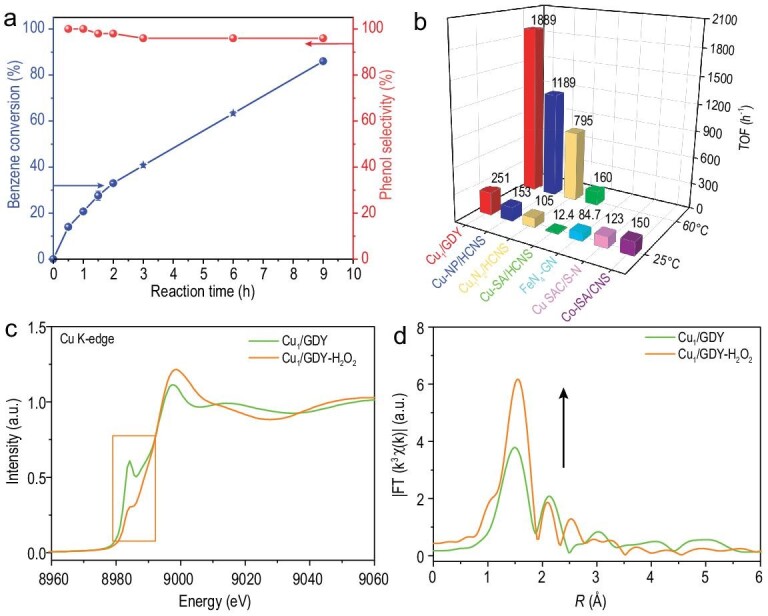
(a) Conversion and selectivity vs. reaction time curves of Cu_1_/GDY, for benzene oxidation to phenol with H_2_O_2_. (b) TOF comparison of Cu_1_/GDY and other metal SACs. (c) Cu K-edge XANES spectra of Cu_1_/GDY and Cu_1_/GDY after H_2_O_2_ treatment. (d) Corresponding FT k^3^-weighted χ(k)-function of the EXAFS spectra.

Direct oxidation of benzene with H_2_O_2_ at 60°C was first conducted to evaluate the catalytic performance of the as-prepared Cu_1_/GDY. Cu_1_/GDY exhibited excellent performance with 21% benzene conversion and 99.9% phenol selectivity after the initial 1 h reaction time (Fig. 3a). The TOF was calculated to be ∼1889 h^−1^, which is significantly higher than the state-of-the-art catalysts reported in the literature under the same reaction conditions (Fig. 3b and Table S2). When the reaction time is increased to 9 h, 86% benzene conversion and 96% phenol selectivity can be obtained. Gas chromatograph-mass spectrometer (GC-MS) analysis identifies the by-product as benzoquinone, which is caused by excessive phenol oxidation and is consistent with previous reports [4,18]. For comparison, only 3% benzene conversion was observed with pristine GDY, suggesting Cu single atoms are the active sites for this reaction.

Because various metal SACs were tested at room temperature for this reaction, the reaction was also conducted at 25°C with Cu_1_/GDY to further compare their catalytic activity. Cu_1_/GDY achieved 7% benzene conversion and 99.9% phenol selectivity in the first 3 h at room temperature. The calculated TOF is ∼251 h^−1^, which is significantly higher than the TOFs reported for catalysts under the same reaction conditions (Fig. 3b and Table S2). Cu_1_/GDY demonstrated excellent recyclability in addition to high activity and phenol selectivity. After the fifth reuse, there was only a slight decrement in benzene conversion (Fig. S6). The TEM image, AC-HAADF-STEM image and EXAFS spectrum (Fig. S7) all show that Cu species remain atomically dispersed after reaction and no Cu-Cu aggregation is formed, demonstrating the stability of Cu single atoms on Cu_1_/GDY, which can be ascribed to the strong interactions between Cu single atoms and the GDY support as discussed above. In addition, the copper content was maintained at ∼0.19 wt% after cycles, suggesting no obvious loss of copper. The slight decrement of activity could be ascribed to the loss of the catalyst during each cycle. Raman and XPS spectra results (Fig. S8) also show that oxidation is not increased by much while the skeleton of GDY is maintained.

It is very impressive that phenol selectivity can be maintained as high as 96% with high benzene conversion at 60°C with Cu_1_/GDY. Wettability matches may play a significant role in such high selectivity. The wettability of a catalyst's surface has a significant impact on the adsorption and desorption behaviors of substrates, and thus on catalytic performance [33]. We test the contact angle (CA) of benzene/phenol on Cu_1_/GDY after H_2_O_2_ treatment. As shown in Fig. S9a, it exhibits a benzene CA of 0°, suggesting the surface of Cu_1_/GDY-H_2_O_2_ is oleophyllic and is very beneficial for benzene adsorption. Because phenol is solid at room temperature and can be dissolved well in water, we test the contact angle of water containing phenol. As shown in Fig. S9b, it exhibits a CA of 119°, suggesting the surface of Cu_1_/GDY-H_2_O_2_ is hydrophobic, and thus is beneficial for phenol desorption. In addition, we did further DFT calculations to investigate the adsorption energies of benzene and phenol on active center O-Cu_1_-C_4_. As shown in Fig. S9c and d, the adsorption energies of benzene and phenol on O-Cu_1_-C_4_ are −3.51 eV and −0.69 eV, respectively. This result indicates benzene is easily adsorbed on O-Cu_1_-C_4_ and the produced phenol is easily desorbed. Non-polar benzene molecules preferentially adsorb on the surface of Cu_1_/GDY during the direct oxidation of benzene to phenol reaction. Once polar phenol is formed, it would be desorbed quickly from the catalyst due to the surface hydrophobic feature and the competitive adsorption of benzene. All the above features suggest Cu_1_/GDY is a fascinating catalyst for efficient catalytic oxidation of benzene to phenol with H_2_O_2_ under mild conditions.

### Reaction mechanism

**Figure 4.**
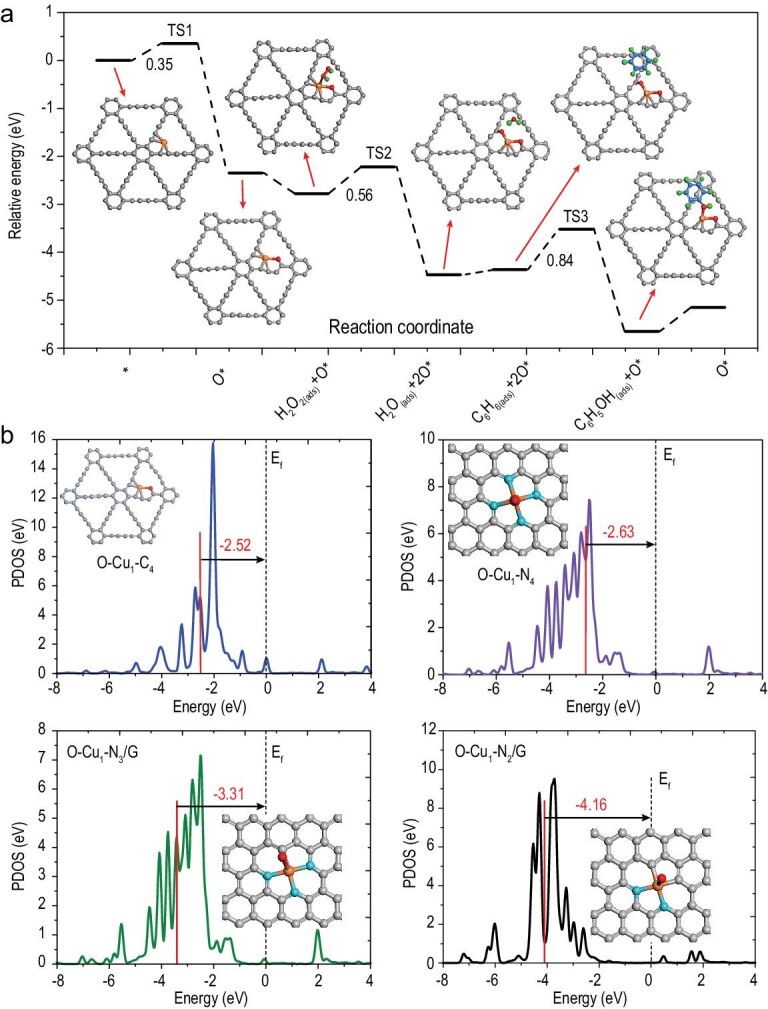
(a) Proposed mechanism of H_2_O_2_ activation and benzene oxidation as produced by DFT calculations of the Cu_1_/GDY catalyst. (b) The *d*-band positions of Cu single atoms in different catalysts as produced by DFT calculations.

To obtain further insights into the high activity of Cu_1_/GDY for benzene oxidation with H_2_O_2_, we performed DFT calculations to investigate the reaction mechanism. Figure 4a shows the free energy profile and reaction pathway on Cu_1_/GDY. In general, the reaction is thought to occur in two steps: (i) the initial formation of activated oxygen species O* by the decomposition of H_2_O_2_; and (ii) the subsequent oxidation of benzene to phenol by the activated oxygen species O* [15,20]. The catalytic reaction begins with the adsorption of an H_2_O_2_ molecule on the Cu single atomic site, which is then easily dissociated by forming a Cu=O intermediate and releasing one water molecule. This process only needs to overcome a low barrier of 0.35 eV and is highly exothermic by −2.35 eV. After that, another H_2_O_2_ molecule is absorbed and further dissociated on the oxidized Cu single site, and then an O=Cu=O center is formed. This process is also energetically favorable, with a moderate energy barrier of 0.56 eV. The generated O=Cu=O center could enable activated oxygen species O* to adopt the benzene molecule via the C−O bonding, and is subsequently converted to phenol directly, with a total energy barrier of 0.84 eV. Meanwhile, this process is also highly exothermic by −1.29 eV. The Cu=O site is regenerated at the end of one reaction after the phenol is released from the active site and participates in the next cycle. The entire reaction pathway is discovered to be exothermic, which is advantageous for mild reaction conditions.

The formation of Cu=O intermediates on Cu_1_/GDY during the reaction was also evidenced by XAFS analysis. As shown in Fig. 3c, compared with the Cu K-edge XANES spectrum of original Cu_1_/GDY, the fingerprint peak (in the orange box) after the H_2_O_2_ treatment decreases and broadens, resulting in loss of D_4h_ symmetry of Cu_1_-C_4_ according to the literature. The EXAFS of Cu K-edge further confirms that the amplitude of the first strong peak in the Fourier-transformed k^3^-weighted plot is significantly enhanced after the H_2_O_2_ treatment (Fig. 3d), suggesting that the coordination number of Cu atoms sharply increases. According to the EXAFS fitting results, the average coordination number increases from 4.0 to 5.2 after the H_2_O_2_ treatment (Table S1). Raman and XPS analysis also support the possibility of C=O formation. As shown in Fig. S8, the total area of C−O and C=O, as well as the intensity ratio of I_D_/I_G_, increase after H_2_O_2_ treatment, which should be attributed to butadiyne carbon oxidation in the GDY skeleton. We further performed Fourier transform infrared spectroscopy (FT-IR) characterization of fresh, H_2_O_2_ treatment and used Cu_1_/GDY. As shown in Fig. S10, an obvious peak at around 640 cm^−1^ corresponding to Cu-O is observed in the FT-IR spectra of H_2_O_2_ treatment and used Cu_1_/GDY. Furthermore, the DFT calculation (Fig. S11) shows that the O-Cu_1_-C_4_ single site is much more stable than the Cu_1_-C_4_ single site in GDY. These findings support the idea that the Cu_1_-C_4_ single site can effectively activate H_2_O_2_ to form Cu=O bonds with more stable intermediate O-Cu_1_-C_4_ single site in GDY, which is an important active intermediate for benzene oxidation to phenol. Similar active intermediates have been proposed and confirmed in other metal SACs with nitrogen coordination structures in the literature [15,16,18,20].

In order to clarify the intrinsic higher activity of Cu_1_/GDY compared with other Cu SACs with nitrogen coordination structures, the *d*-band positions of Cu single atoms in O-Cu_1_-C_4_/GDY and O-Cu_1_-Nx/G (x = 2, 3, 4) are investigated. Generally, the closer the *d*-center of metal atoms is to Fermi level, the higher the catalytic activity is. As shown in Fig. 4b, the Cu-3*d* band center in O-Cu_1_-C_4_/GDY is calculated to be −2.52 eV, much closer to Fermi level than that in O-Cu_1_-N_2_/G (−4.28 eV), O-Cu_1_-N_3_/G (−2.81 eV) and O-Cu_1_-N_4_/G (−2.63 eV). Therefore, Cu_1_/GDY exhibits the highest catalytic activity.

## CONCLUSIONS

In summary, we developed an efficient method of producing a GDY-supported Cu single-atom catalyst with uniform Cu_1_-C_4_ coordination structure by making use of the strong d-π interaction between Cu atoms and diacetylenic. When compared to state-of-the-art SACs for direct benzene oxidation to phenol with H_2_O_2_, the as-prepared Cu_1_/GDY demonstrated significantly higher activity. Furthermore, due to the hydrophobic and oleophyllic surface nature of Cu_1_/GDY, which is beneficial for benzene adsorption and phenol desorption, high phenol selectivity was obtained under high benzene conversion. Both experiments and DFT calculations indicated that the high activity was due to the formation of O-Cu_1_-C_4_ active intermediates in GDY, which has a *d*-band center position that is closer to the Fermi level. This work not only presents a facile and efficient route for fabricating GDY-supported metal SACs with uniform metal-C_4_ centers, but also provides a promising benzene hydroxylation catalyst for phenol production with H_2_O_2_.

## Supplementary Material

nwac018_Supplemental_File
