# Supplementary material for: Uniform single atomic Cu1-C4 sites anchored in graphdiyne for hydroxylation of benzene to phenol
Source: Natl Sci Rev. 2022 Feb 11;9(9):nwac018. doi: 10.1093/nsr/nwac018 (PMC9584062; doi:10.1093/nsr/nwac018)
Supplement: nwac018_Supplemental_File [file nwac018_supplemental_file.doc]

**Supplementary Information**

**Uniform Single Atomic Cu_1_-C_4_ Sites Anchored in Graphdiyne for** **Hydroxylation of Benzene to Phenol**

Jia Yu,^1, 3^ Changyan Cao*,^1, 3^ Hongqiang Jin,^1, 3^ Weiming Chen,^1, 3^ Qikai Shen,^1, 3^ Peipei Li,^1, 3^ Lirong Zheng,^4^ Feng He*,^2^ Weiguo Song^1, 3^ and Yuliang Li^2, 3^

^1^  Beijing National Laboratory for Molecular Sciences, CAS Research/Education Center for Excellence in Molecular Sciences, Laboratory of Molecular Nanostructures and Nanotechnology, Institute of Chemistry, Chinese Academy of Sciences, Beijing 100190, China

E-mail: [cycao@iccas.ac.cn](mailto:cycao@iccas.ac.cn;)

^2^  Beijing National Laboratory for Molecular Sciences, CAS Research/Education Center for Excellence in Molecular Sciences, Laboratory of Organic Solids, Institute of Chemistry, Chinese Academy of Sciences, Beijing 100190, China

E-mail: [hefeng2018@iccas.ac.cn](mailto:hefeng2018@iccas.ac.cn)

^3^ University of Chinese Academy of Sciences, Beijing 100049, China

^4^ Beijing Synchrotron Radiation Facility (BSRF), Institute of High Energy Physics, Chinese Academy of Sciences, Beijing 100049, China

**Chemicals:** Tetrabutylammonium fluoride 1 M in THF (TBAF), Copper(II) acetate (Cu(OAc)_2_), anhydrous Zinc chloride, benzene were purchased from Alfa Aesar. Hexabromobenzene was brought from J&K Scientific. Tetrakis (triphenylphosphine) palladium, n-Butyllithium and Trimethylsilylacetylene were bought from Energy Chemical. Hydrogen peroxide (30 wt%) was purchased from Sinopharm Chemical Reagent Co.,Ltd Toluene and tetrahydrofuran (THF) were re-fluxed with the Na under N_2_ atmosphere for sufficient time in order to remove trace amount of water. All reagents and solvents were used without further purification unless specifically mentioned.

**Synthesis of Cu_1_/GDY:** Hexakis[(trimethylsilyl) ethynyl]benzene (HEB-TMS) was first prepared according to the reported method (*Chem. Commun*. 2010; 46: 3256-8; *Chem. Commun*. 2017; 53: 8074-7). 2 mL TBAF (2 mmol, 1 M in THF) was added to the solution of HEB-TMS (131 mg, 0.198 mmol, in 50 mL THF) and stirred at 0 °C for 30 min under inert atmosphere to remove the protecting group. Then, the solution was washed with saturated sodium chloride solution for three times and dried by anhydrous Na_2_SO_4_. The HEB solution was then diluted with 20 mL THF. Then proper amount of Cu(OAc)_2_ dissolved in 1 mL THF was added dropwise to the HEB solution, and stirred at room temperature for 1 h. All the above operations should be carried out quickly under dark conditions. The solvent was removed under vacuum and was obtained.

Subsequently, the powder was transferred little by little to a beaker pre-heated to 120 °C in the air. The ultra-fast reaction accompanied by a slight explosion was occurred in seconds, and the color was changed from pale yellow to black, suggesting the successful synthesis of Cu_1_/GDY.

**Catalytic performance test**

The hydroxylation of benzene reaction was performed in a 100 mL round-bottom glass flask. 10 mg catalyst (Cu_1_/GDY, GDY), 0.3 mL benzene, 5 mL H_2_O_2_ (30 wt%), and 6 mL CH_3_CN was added. The reactor was sealed and reacted at 25 °C, 30 °C, 45 °C and 60 °C for different time, respectively. (*Caution: H_2_O_2_ is explosive and much O_2_ gas is generated. The reaction should be done in the fume hood. The reaction volume should be less than 1/5.)* After the system was cooled down to room temperature, the mixture was extracted with ethyl acetate. The extraction solution was analyzed with GC−MS and GC with n-heptadecane as an internal standard. General procedure for recyclability test: After each reaction, the catalyst was separated from the reaction system by centrifugal separator. Then the collected catalyst was washed by ethyl acetate three times, was used in the next batch of catalytic reaction at 60 °C for 1 h, immediately. The phenol selectivity was denoted as the percentage of the desired phenol relative to the total oxidation products composed of the desired phenol and the byproducts quinone. In all cases, only quinone as byproduct was detected.

TOF was obtained as follow:

TOF (h^-1^) = benzene conversion (mol) / [Cu active sites (mol) x reaction time (h)]

TOFs values were calculated and based on the total Cu loading in the catalysts.

**Characterizations**

The powder XRD patterns were collected using a Rigaku D/max 2500 diffractometer equipped with Cu Kα radiation (λ = 1.5418 Å) at 40 kV and 200 mA. Raman spectrum were recorded on a the DXR Raman by using the 532 nm line of an Argon ion laser as the excitation source. X-Ray photoelectron spectra were acquired on the VG Scientific ESCALab220i-XL spectrometer using 300W Al Kα radiation. The morphology and microstructures of the samples were measure on the transmission electron microscopy (TEM) (JEM-2100F, JEOL, Japan), high-resolution transmission electron microscopy (HRTEM) (JEM-2100F, JEOL, Japan) working at 200 kV, the field emission scanning electron microscopy (FESEM) (HITACHI S-4800, Japan) working at 10 kV. Secondary electron images was obtained using a FEG JOEL F200 (JEOL Ltd.) operating at 200 kV acceleration voltage in STEM mode using a SEI detector. Element mapping was characterized on HRTEM equipped with Oxford detection. Aberration-corrected HAADF-STEM measurements were performed with a cold-FEG aberration-corrected JEOL Grand ARM 300 (JEOL Ltd.) operating at 300 kV acceleration voltage. The instrument is equipped with double Cs-correctors. The microscope in STEM mode have two annular dark field (ADF) detectors and an annular bright field (ABF) detector. Inductively coupled plasma atomic emission spectroscopy (ICP-AES, Shimadzu ICPE-9000) was used to measure the loading content of copper on the catalysts. The Brunauer–Emmett–Teller (BET) surface areas of the samples were characterized by measuring the N_2_ adsorption and desorption isotherms on a Micromeritics ASAP 2460 surface area and porosity analyzer at 77 K. Before the BET measurement, all samples were degassed under dynamic vacuum at 220°C for 12 h.

**X-ray absorption spectroscopy (XAS) measurements and analysis**

The X-ray absorption find structure spectra (Cu K-edge) were collected at 1W1B station in Beijing Synchrotron Radiation Facility (BSRF). The storage rings of BSRF was operated at 2.5 GeV with an average current of 250 mA. Using Si(111) double-crystal monochromator, the data collection were carried out in transmission/fluorescence mode using ionization chamber. All spectra were collected at room temperature in ambient conditions. The acquired EXAFS data were processed according to the standard procedures using the ATHENA module implemented in the IFEFFIT software packages. The k^3^-weighted EXAFS spectra were obtained by subtracting the post-edge background from the overall absorption and then normalizing with respect to the edge-jump step. Subsequently, k^3^-weighted χ(k) data of Fe K-edge were Fourier transformed to real (R) space using a hanning windows (d_k_=1.0 Å^-1^) to separate the EXAFS contributions from different coordination shells. To obtain the quantitative structural parameters around central atoms, least-squares curve parameter fitting was performed using the ARTEMIS module of IFEFFIT software packages.

**DFT Computations**

All calculations were carried out by employing density functional theory (DFT), as implemented in the Vienna Ab-initio Simulation Package. The projector augmented-wave pseudopotentials and a cutoff energy of 400 eV for the plane-wave basis set were adopted. The generalized gradient approximation (GGA) method with spin polarized Perdew-Burke-Ernzerhof (PBE) functionals was used to describe the exchange-correlation potential. The Brillouin zone integrations were sampled using the G points Monkhorst-Pack grid for the systems. The Cu/GDY single atom catalyst model was embedded in a periodic 2 × 2 supercell of graphdiyne support (72 carbon atoms). To avoid the interlayer interactions, the vacuum thickness between graphdiyne layers was set as 15 Å along the surface normal. Transition states were searched using the climbing image nudged elastic band (CI-NEB) method. All atoms were relaxed to their equilibrium positions when the energy was converged to 1×10^−5^ eV, and the forces were converged to 0.03 eV Å^−1^. The free energies (G) of the reactants, surface intermediates, and products were obtained using the equation G = E_total_ + ZPE − TS, where E_total_ is the total energy of the species, ZPE is the zero-point energy, and S is the entropy.


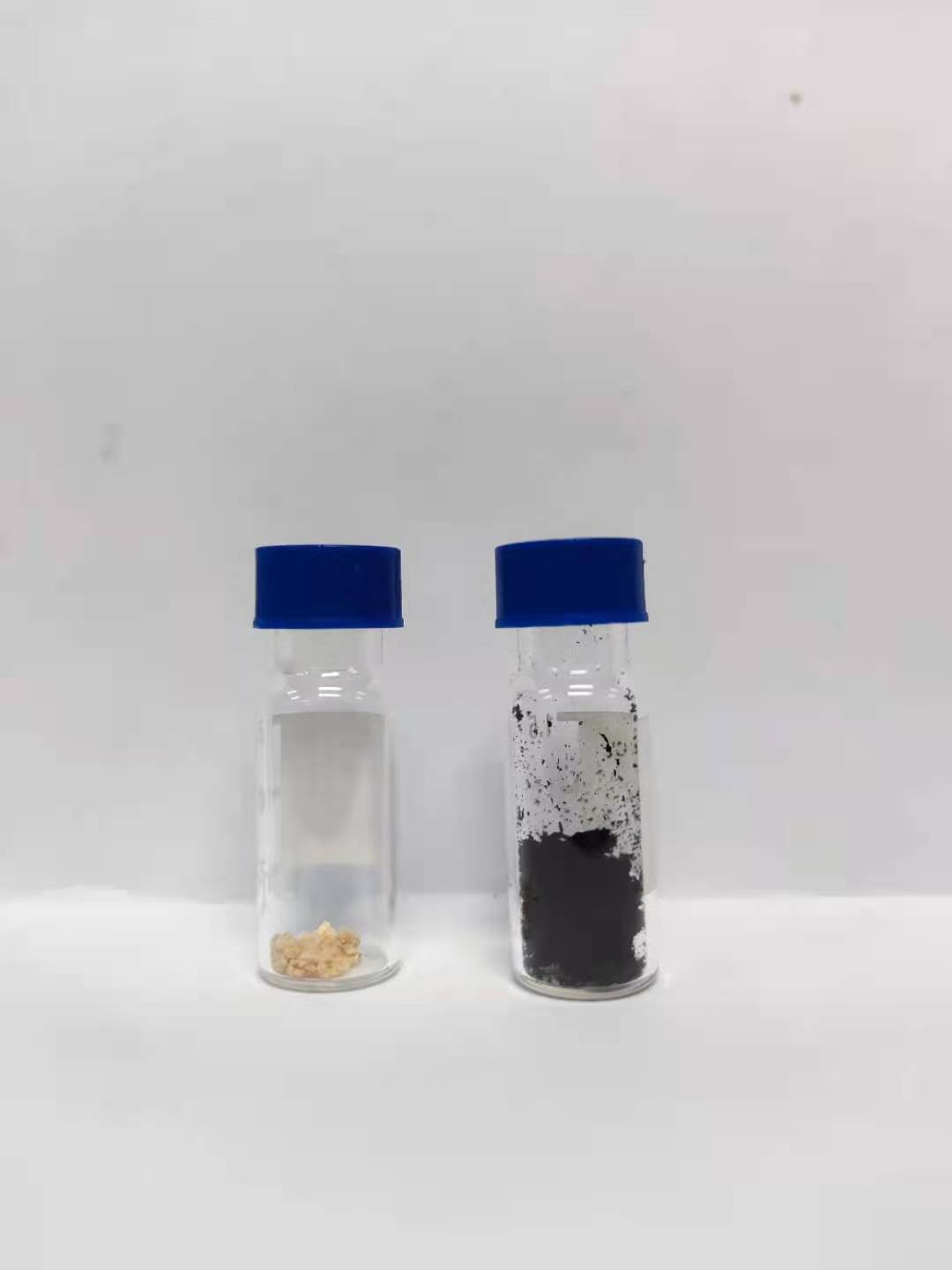


**Figure S1.** Photographs of (left) HEB monomer and Cu ion compounds, (right) the obtained black Cu_1_/GDY after heating the compounds.

**Figure S2.** (a) low-magnification SEM image, (b) TEM image, (c-d) HRTEM images of Cu_1_/GDY.

**Figure S3.** XRD pattern of Cu_1_/GDY.

**Figure S4.** Cu 2p XPS spectrum of Cu_1_/GDY.

**Figure S5.** N_2_ adsorption-desorption isotherms of (a) Cu_1_/GDY and (b) pure GDY.


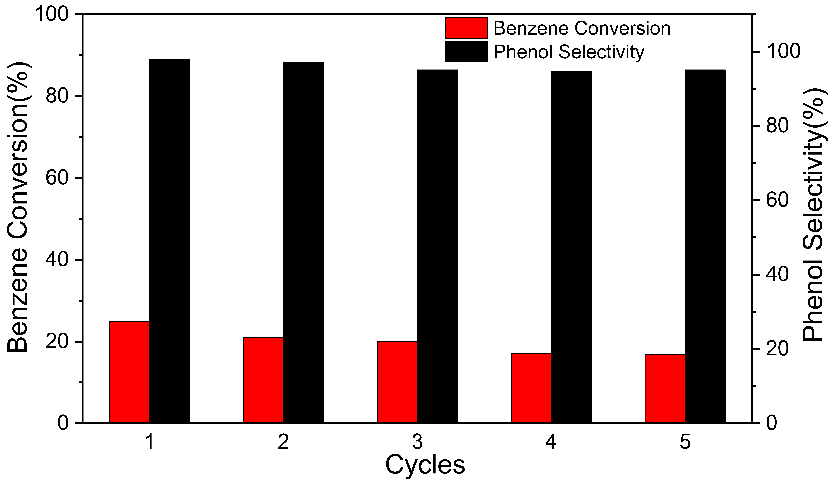


**Figure S6.** Recycle performance of Cu_1_/GDY catalyst for benzene oxidation. Reaction conditions: 10 mg catalyst, 0.3 mL benzene, 5 mL H_2_O_2_ (30%), and 6 mL CH_3_CN at 60 °C for 1h.

**Figure S7.** (a) STEM-EDS mapping images, (b) HAADF-STEM images, (c) Normalized Cu K-edge XANES spectrum and (d) the corresponding EXAFS fitting curve of the recycled Cu_1_/GDY.


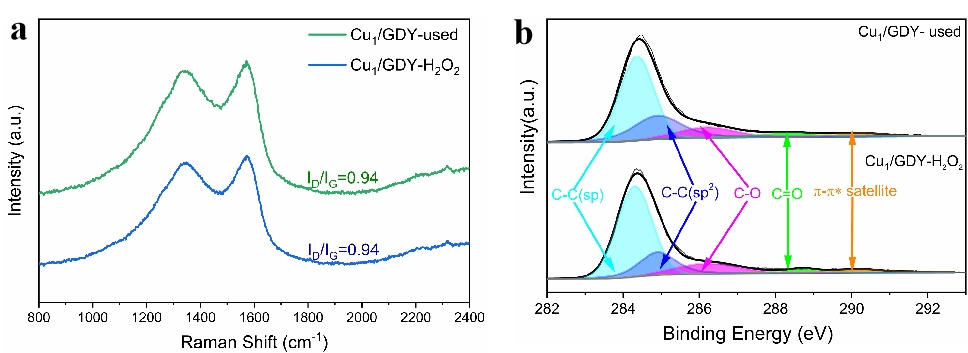


**Figure S8.** (a) Raman spectrum and (b) High resolution C 1s XPS spectrum of recycled Cu_1_/GDY.


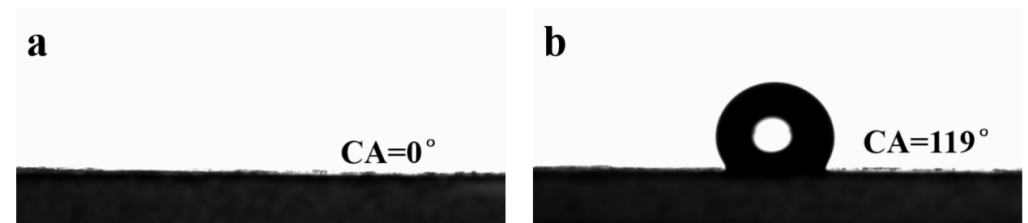


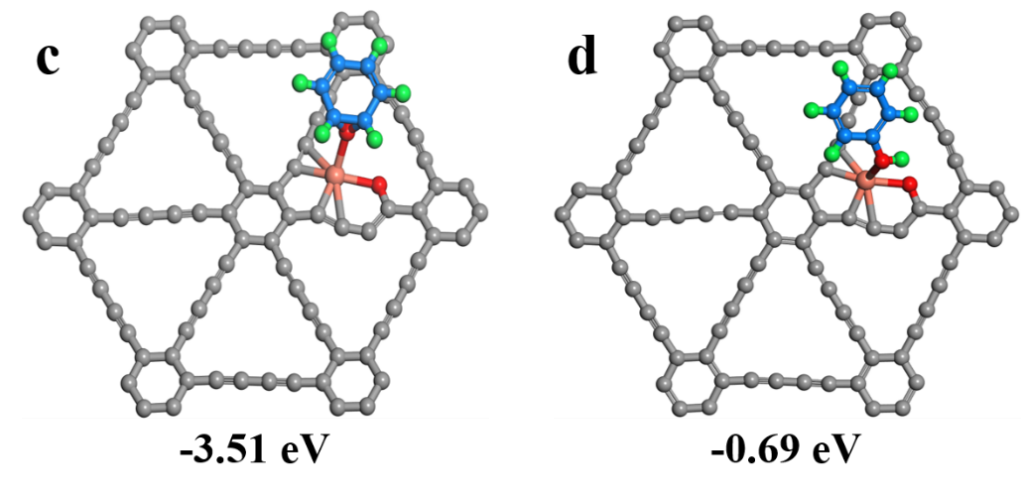


**Figure S9.** (a) static benzene and (b) static phenol in water Contact angle (CA) measurements on Cu_1_/GDY-H_2_O_2_. (c-d) The absorption energies of benzene and phenol on O-Cu_1_-C_4_ by DFT calculations.

**
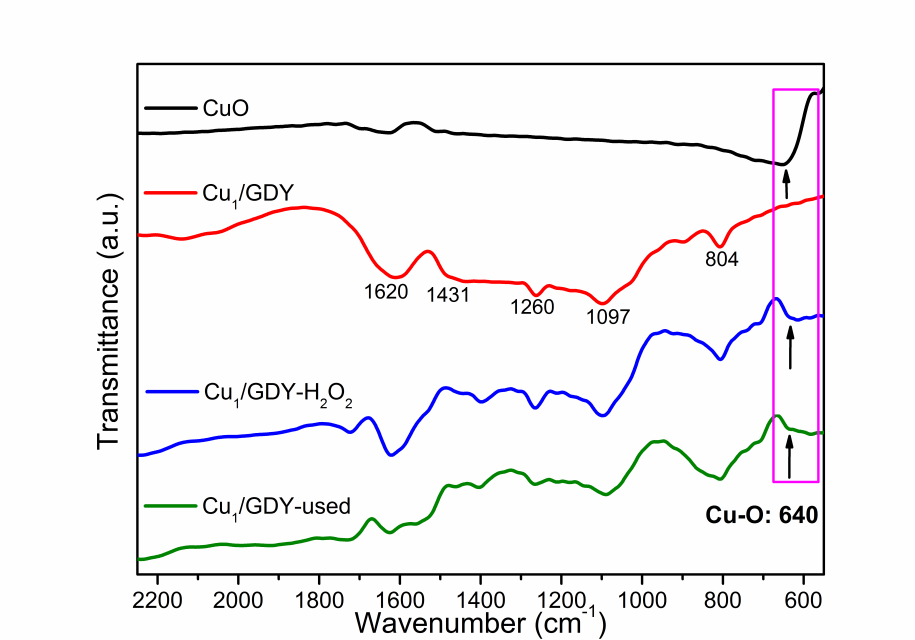
**

**Figure S10.** FT-IR spectra of Cu_1_/GDY, Cu_1_/GDY after H_2_O_2_ treatment, Cu_1_/GDY after used and CuO standard sample.


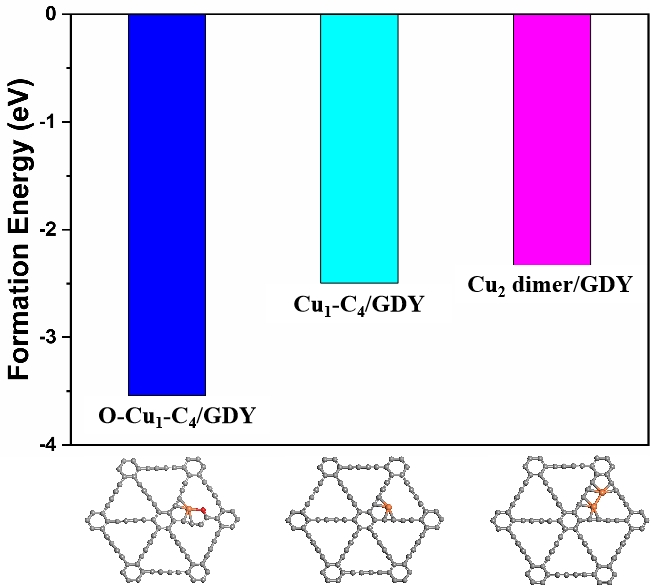


**Figure S11.** Formation energies of O-Cu_1_-C_4_/GDY, Cu_1_-C_4_/GDY and Cu_2_ dimer/GDY by DFT calculations.

**Table S1.** EXAFS fitting parameters at the Cu K-edge for various samples

| Sample | Shell | N ^a^ | R (Å) ^b^ | σ^2^ (Å^2^·10^-3^) ^c^ | ΔE_0_ (eV) ^d^ | *R* factor (%) |
| --- | --- | --- | --- | --- | --- | --- |
| Cu_1_/GDY | Cu-C(sp) | 4.0 | 1.92 | 8.4 | 4.6 | 0.7 |
|  | Cu-C(sp^2^) | 2.5 | 2.64 | 9.9 |  |  |
| Cu_1_/GDY after H_2_O_2_ treatment | Cu-C(sp) | 5.2 | 1.97 | 6.7 | 6.1 | 0.4 |
|  | Cu-C(sp^2^) | 2.5 | 2.61 | 6.8 |  |  |
| Cu_1_/GDY  after reused | Cu-C(sp) | 6.2 | 1.96 | 6.2 | -4.4 | 0.7 |
|  | Cu-C(sp^2^) | 2.5 | 2.57 | 7.1 |  |  |

*^a^* *N*: coordination numbers; *^b^* *R*: bond distance; *^c^* *σ*^2^: Debye-Waller factors; *^d^* Δ*E*_0_: the inner potential correction. *R* factor: goodness of fit. *Ѕ*02 was set as 0.90 for Cu-C.

Notes: errors of N、*σ*^2^: 20%, Accuracy of R: ± 0.03 Å

**Table S2.** Catalytic performances comparison of Cu_1_/GDY and other catalysts

| **Catalyst** | **Reaction Condition** | **T (°C)** | **Time (h)** | **Conversion (%)** | **Phenol Selectivity (%)** | **TOF(h^-1^)** | **Reference** |
| --- | --- | --- | --- | --- | --- | --- | --- |
| **Cu_1_/GDY** | **10 mg cat. 0.3ml benzene** | **60** | **9** | **86** | **96** | **1889 (1 h)** | **This work** |
|  |  | **25** | **3** | **7** | **99.9** | **251 (3 h)** |  |
| Cu_1_-N_2_/HCNS | 50 mg cat. 0.4ml benzene | 60 | 3.5 | 89.3 | 90.5 | 795 (3.5 h) | iScience 2019, 22, 97-108. |
|  |  | 25 | 24 | 81.2 | 86.6 | 105 (3.5 h) |  |
| Cu-SA/HCNS | 50 mg cat. 0.4ml benzene | 60 | 12 | 86 | 96.7 | 160 (1 h) | J. Am. Chem. Soc. 2018, 140, 16936-16940 |
| Cu-SA/HCNS | 50mgcat. 0.4ml benzene | 25 | 24 | 33.4 | 90.6 | 12.4 (1 h) | J. Am. Chem. Soc. 2018, 140, 16936-16940 |
| Cu-NP/HCNS | 50mgcat. 0.4ml benzene | 25 | 24 | 41.2 | 64.2 | 153 (1 h) | J. Am. Chem. Soc. 2018, 140, 16936-16940 |
| VO-peg-C_3_N_4_ | 75 mg cat. 1ml benzene | 60 | 8 | 19.6 | 97.1 | / | Applied Catalysis A: 2018, 549, 31-39 |
| SA-Fe/CN | 20mgcat. 0.5ml benzene | 60 | 24 | 45 | 94 | / | J. Am. Chem. Soc. 2017, 139, 10976-10979 |
| ISAS Fe/NPC | 50 mg cat. 0.1ml benzene | 60 | 24 | 42.6 | 99 | / | Chem. Commun., 2020,  56, 8916 |
| CCG | 50 mg cat. 0.15ml benzene | 60 | 16 | 18 | 99 | / | EnergyEnviron.Sci.,2013,6, 793-798 |
| Cu SAC/S-N | 25 mg cat. 0.2 ml benzene | 25 | 48 | 60.2 | 85 | 123 (1 h) | J. Am. Chem. Soc. 2020, 142, 12643-12650 |
| FeN_4_-GN | 50 mg cat. 0.4ml benzene | 25 | 24 | 23.4 | 79.9 | 84.7 (5 min) / 27.7 (1 h) | Sci Adv 1 (11), e1500462. |
| Co-ISA/CNS | 50 mg cat. 0.4ml benzene | 25 | 96 | 68 | 90 | 150 (1 h) | Nat Commun 9, 3861 (2018) |
| Fe-N_4_/N-C | 50 mg cat. 0.4ml benzene | 30 | 24 | 78.4 | 100 | / | Nat Commun 10, 4290 (2019) |
